# Reversal of a Spatial Discrimination Task in the Common Octopus *(Octopus vulgaris)*

**DOI:** 10.3389/fnbeh.2021.614523

**Published:** 2021-06-25

**Authors:** Alexander Bublitz, Guido Dehnhardt, Frederike D. Hanke

**Affiliations:** ^1^Sensory and Cognitive Ecology, Institute for Biosciences, University of Rostock, Rostock, Germany; ^2^Neuroethology, Institute for Biosciences, University of Rostock, Rostock, Germany

**Keywords:** spatial learning, cognitive abilities, behavioral plasticity, cognition, cognitive flexibility

## Abstract

Reversal learning requires an animal to learn to discriminate between two stimuli but reverse its responses to these stimuli every time it has reached a learning criterion. Thus, different from pure discrimination experiments, reversal learning experiments require the animal to respond to stimuli flexibly, and the reversal learning performance can be taken as an illustration of the animal's cognitive abilities. We herein describe a reversal learning experiment involving a simple spatial discrimination task, choosing the right or left side, with octopus. When trained with positive reinforcement alone, most octopuses did not even learn the original task. The learning behavior changed drastically when incorrect choices were indicated by a visual signal: the octopuses learned the task within a few sessions and completed several reversals thereby decreasing the number of errors needed to complete a reversal successively. A group of octopus trained with the incorrect-choice signal directly acquired the task quickly and reduced their performances over reversals. Our results indicate that octopuses are able to perform successfully in a reversal experiment based on a spatial discrimination showing progressive improvement, however, without reaching the ultimate performance. Thus, depending on the experimental context, octopus can show behavioral flexibility in a reversal learning task, which goes beyond mere discrimination learning.

## Introduction

A reversal learning experiment is a classic experiment to investigate the cognitive abilities of an individual and was originally used by Bitterman and colleagues to compare the learning abilities of different species (Bitterman, 1965). Studying the cognitive abilities of *Octopus vulgaris* is of particular interest, as this species, although belonging to the mollusks, is usually considered to possess extraordinary or “vertebrate-like” cognitive abilities (Mather et al., 2010) such as its ability to learn from observing conspecifics (Fiorito and Scotto, 1992). Moreover, high cognitive abilities are often inferred from the large size and organization of its brain (Young, 1971).

During a reversal learning task, the animal first has to learn to discriminate between two stimuli to a predefined criterion during the acquisition phase. After passing the criterion, the signs of the stimuli are reversed, and the previously positive stimulus is now changed into the negative stimulus and vice versa. Usually, a number of reversals are conducted to test whether the animal will show progressive improvement in such a serial reversal learning experiment; progressive improvement is defined as a decrease in the number of errors per reversal over all reversals conducted. Some animals even achieve the ultimate performance of one-trial learning; they need to experience only one error to realize that a reversal has taken place (see, for example, Mackintosh and Mackintosh, 1964; Balderrama, 1980; Karson et al., 2003). Thus, over time, some animals learn to learn (Harlow, 1949); they are forming a reversal learning set. This learning ability illustrates that reversal learning is going beyond mere discrimination learning during which an animal learns stimulus specific responses (Shettleworth, 1998). Reversal learning has usually been considered a good indicator for behavioral flexibility. Species, especially those that inhabit complex environments, profit from behavioral flexibility, as it will allow them to adapt to changes in their environment and/or to find suitable alternatives quickly (Day et al., 1999; Bond et al., 2007; Lea et al., 2020).

Just recently, Bublitz et al. (2017) revisited visual reversal learning in octopus in good tradition of work of the mid-twentieth century (Boycott and Young, 1957; Mackintosh, 1962; Young, 1962b; Mackintosh and Mackintosh, 1963, 1964). These previous studies analyzed various aspects of visual reversal learning such as the effect of reversing daily without prior reaching a learning criterion, the effect of overtraing on reversals with and without irrelevant cues, or the effect of vertical lobe removal on reversal learning (for overview see Table 1 in Bublitz et al., 2017) with the vertical lobe being an essential neuronal structure for learning and memory (see for example, Young, 1960, 1970). From these studies, it was concluded that octupus can perform multiple reversals and can increase its performance over reversals. Bublitz et al. (2017) refined the general methodological approach of the previous visual reversal learning studies by the elimination of pretraining or the introduction of a secondary reinforcer, thereby conducting a “classic” visual serial reversal learning experiment. The results varied considerably between individuals. One of the individuals participating in the study of Bublitz et al. (2017) showed a very good reversal learning performance, reducing the number of errors over four completed reversals. In contrast, the three other individuals failed to reach the learning criterion already during the first or second reversal. Moreover strong stimulus preferences occurred that might have affected learning in general and reversal learning in particular.

In many animals, the performance in a visual reversal learning experiment is contrasted with the performance in a spatial reversal learning task in which the individual either has to choose the right or left side, a very simple spatial discrimination, depending on the experimental stage. For a number of animals, the performance in the latter is better than in a visual reversal learning experiment (see, for example, skunks in Doty and Combs, 1969; or painted turtles in Holmes and Bitterman, 1966). In general, good spatial reversal learning performance including progressive improvement has been documented for various species ranging from bumblebees (Chittka, 1998), pigeons (see, for example, Gonzalez et al., 1967; Ploog and Williams, 2010), to dogs (Tapp et al., 2003) and horses (Potter and Fiske, 1979); one-trial learning occurred in chimpanzees (Schusterman, 1964), rats (Dufort et al., 1954), or cockroaches (Balderrama, 1980). One explanation for this phenomenon is related to the fact that the spatial discrimination does not involve irrelevant cues as does the visual task during which the side, left or right, is the irrelevant cue on which the animal should not focus on for making its response. Consequently, in line with these previous studies, octopus might also perform better in spatial discrimination tasks and its reversals. In addition, orientation in space might be a crucial ability for most mobile species, as the octopus, which might consequently result in a better spatial than visual (reversal) performance. Spatial orientation, in general, is also expected to play a major role for octopus, which is a central place forager (Mather, 1991b). The octopus individuals often hide themselves in their dens. However, they leave their shelters to go for foraging. Good spatial abilities are required to return to the den after the foraging trip. These spatial abilities are also asked for if an octopus decides to move into a new den. An additional factor that might assert even more pressure on the development of good spatial orientation skills is that octopus is a soft-bodied animal that faces considerable predatory pressure (Mather and O'Dor, 1991). Thus, reducing the amount of time outside the shelter to a minimum by good spatial knowledge seems to be critical for survival. Besides these theoretical considerations deduced from the octopus ecology, experimental evidence for good spatial skills is already available for octopus: octopus species have been shown to rely on landmarks for spatial orientation (Mather, 1991b), some individuals successfully performed in detour experiments (Wells, 1964, 1967, 1970), and they were able to (re)locate burrows in arenas (Boal et al., 2000). Moreover indirect evidence for good spatial skills results from the observations of Mather (1991a) describing *O. vulgaris* as often moving to new places in successive hunts, which again requires spatial knowledge to be able to return to the den.

Among the cephalopods, spatial reversal learning has only been addressed in *O. maya* (Walker et al., 1970) and *Sepia officinalis* (Karson et al., 2003). In Walker et al. (1970), however, the signs of the stimuli were only reversed twice, and finally, training was stopped at the beginning of the second reversal. In contrast, Karson et al. (2003) conducted a classic spatial serial reversal learning experiment with common cuttlefish in which one individual even completed eight reversals, and two individuals met the learning criterion with one or two errors.

Spatial serial reversal learning has not been tested in *O. vulgaris* yet, our model species for cognitive abilities. Thus, the main goal of this study was to collect data on spatial reversal learning in *O. vulgaris* to further elucidate on the reversal learning abilities of octopus as a measure of their cognitive flexibility. This data set might also allow comparing the visual and spatial reversal performance of octopus. We hypothesized that octopus performs better in the spatial serial reversal learning experiment, as (1) stimulus preferences dominating visual discrimination experiments do not play a role in spatial tasks, and as (2) there is good theoretical as well as empirical evidence that octopus possesses good spatial abilities.

During training of the original task, three octopus individuals failed to improve their performances. In an attempt to overcome stagnation, we introduced an incorrect-choice signal (ICS), presented after an incorrect response. As after its introduction, the octopus individuals easily reached the learning criterion and could complete several reversals successfully, we set out to study the effect of this ICS. Thus, we trained a second group of octopus individuals without prior training experience without ICS and compared the learning performance of these two groups.

## Materials and Methods

### Experimental Subjects

Seven subadult *O. vulgaris* (Ov1–Ov7), caught in the Tuscan Archipelago of the Mediterranean Sea, served as experimental subjects. The number of experimental animals compares well with the sample size of previous reversal learning studies including capuchin monkeys, turtles, lizards, crayfish, minks, ferrets, and skunks (Holmes and Bitterman, 1966; Capretta and Rea, 1967; Doty and Combs, 1969; Day et al., 1999; Beran et al., 2008). Previous octopus reversal learning studies had trained 4–10 individuals per condition (Boycott and Young, 1957; Mackintosh, 1962; Young, 1962b; Mackintosh and Mackintosh, 1963, 1964). The dorsal mantle length of the octopus of this study was 5–8 cm. Sex could only be determined in Ov5, a male octopus. Except for Ov3, all animals were experimentally naive. Ov3 had already been trained for 1,160 trials to choose one out of two target tubes (TT), the task of this study, however, using two identical optical stimuli moving up and down close to the TTs instead of the monitor lightening up as start signal (see *Experimental setup* and *Experimental procedure* for details). Due to differences in training, the data of Ov3 will only be presented in the Supplementary Material; however, they will not be included in the analyses of this manuscript.

The animals were transported in containers containing natural sea water. After transportation, under continuous monitoring, the animals were adapted to the conditions of the home tanks by adding water from the home tanks to the containers slowly and dropwise before they were inserted in the home tanks.

The animals were kept according to the recommendations on maintenance, care, and welfare given for cephalopods (Smith et al., 2013; Fiorito et al., 2014, 2015). All seven subjects were housed individually in 250-L glass tanks (100 × 50 × 50 cm) with a substrate of sand, coral, stones, and shells that allowed the animals to hide and build a den. The tanks were filled with natural sea water with a salinity of 35 g/kg at a water temperature of 19–23°C. These parameters of maintenance as well as all essential parameters of water quality were regularly checked. With the help of artificial illumination, a natural day/night cycle of 12 h/12 h was achieved.

Food was usually provided to the subjects twice a day exclusively during experiments and according to their performance, however, overall assuring that the animal got an adequate amount of food every day. On days, on which no experiment was conducted, the animals were fed *ad libitum*. The amount of food taken on these days allowed adjusting the amount of food given during experiments to achieve good satiation daily. The experimental animals were fed with either bivalve or gastropod mollusks. The type of food was chosen according to individual preferences as well as availability but was kept constant for one individual over the entire experimental period. Uneaten food was removed after feeding.

Depending on the individual and its motivation, a single experimental session run with one octopus individual lasted from 90 min up to approximately 2 h. Experiments were conducted 5–7 days a week over a total period of up to 7 months of training.

The animals' health status including, for example, its posture, movements, changes in body pattern, vigilance, or feeding behavior were controlled at least every morning and evening. This study was conducted in accordance with the directive 2010/63/EU, and maintenance and the experiments (Permit No. 6712GH00113, Staatliches Amt für Umwelt und Natur Rostock, Landesamt für Landwirtschaft, Lebensmittelsicherheit und Fischerei, Mecklenburg-Vorpommern) as well as transport (EG Verordnung 1/2005, Reg.-Nr. 082120000714) were approved by local authorities.

### Experimental Setup

**Figure 1:**
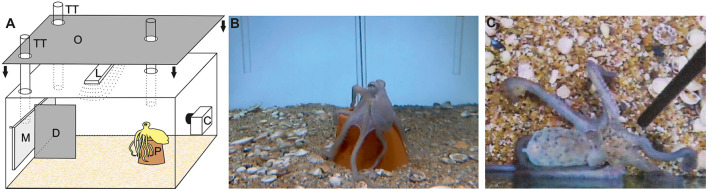
Experimental setup. **(A)** A liquid crystal display (LCD) monitor M was attached to the tank from outside to signal the start and the end of each single trial by lighting and dimming the monitor, respectively. The area in front of the monitor was separated by a divider D into two compartments. In each compartment, a target tube TT was inserted through the lid of the aquarium; the animals were required to touch the target tube for a response. Each single trial started with the animal positioning itself on a flower pot P at approximately 50 cm distance to the monitor. The whole area was illuminated by a lamp L. To avoid secondary cues during experiments, the top of the aquarium and the side walls were shielded with an opaque cover O (side cover not shown for clarity). Experiments were observed and recorded with the help of a camera C. Not drawn to scale. **(B)** Photograph of the camera with which the aquarium could be overseen even if the aquarium was covered from all sides. The octopus is sitting on the flower pot, and the two target tubes are on the left and right side in front of the monitor, which has already turned bright signaling the start of the trial. A third tube close to the flower pot served to lure the octopus to the flower pot, if necessary. Please note that the divider can hardly be seen due to the position of the camera with respect to it and as it is aligned with the third tube. **(C)** After an incorrect response, the octopus was presented with an incorrect choice signal (ICS), a black rod, upon which the octopus initially/sometimes changed their body pattern to a broad mottle pattern—the ICS was introduced when training stagnated (group 1) or right from the start of training (group 2).

The general experimental setup is shown in Figure 1. The components of the experimental setup were installed inside the individual home tanks before starting an experimental session. Outside the tank at one fare end, an LCD monitor was permanently attached (21.5 in., 60 Hz, E2251 Full HD, LG electronics, Inc., Seoul, South Korea). The monitor was lit to signal the start and dimmed to signal the end of a trial. As octopus is polarization sensitive (Hanke and Kelber, 2020), the animals might have used either the polarization and/or the luminance information as start or end signal. A vertical divider separated the area in front of the monitor into equally large left and right compartments, compartments A and B. Within each compartment in the outer right, respectively, the outer left corner and close to the LCD monitor, a transparent acrylic TT (length, 55 cm; diameter, 3 cm) was inserted through the lid of the aquarium. These TTs served as targets that the animals were supposed to touch and provided the food reward to the subjects in case of a correct response. As established by Bublitz et al. (2017), the food reward was preceded by a secondary reinforcer, a transparent acrylic rod with a black tip that was moved up and down the respective tube. Upon an incorrect response, a black plastic rod could be inserted into the aquarium, the incorrect-choice signal (ICS; see *Experimental procedure*, Figure 1C and Supplementary Video 2). At approximately 50 cm distance to the monitor and aligned with the center of the monitor, a terracotta flower pot served as a starting point for each single trial during experiments and ensured that the subjects had approximately the same viewing angle on the display and the TT, subtending 50°, and the same distance to the TTs at the beginning of each trial. A feeding tube inserted right above the terracotta flower pot was used to lure the animal back to the starting point after its response, if necessary. For luring, the secondary reinforcer was gently moved up and down this feeding tube, which usually attracted the octopus' attention.

During experiments, an opaque curtain around the aquarium as well as an opaque cover on the lid of the tank kept the experimenter out of sight of the octopus in order to avoid unintentional secondary cueing. The experimenter observed the experimental procedure via a camera (Genius WideCam 1050, KYE System Corporation 2011, Taipei, Taiwan) equipped with a wide-angle lens. The whole experimental area was illuminated with a lamp from above.

### Experimental Procedure

After the insertion of the animals into the aquaria, they were first allowed to adapt to the new environment. When they started to take food from the experimenter, which usually happened within 1–3 days after insertion into the home tanks, pretraining started, which involved the establishment of the secondary reinforcer (Bublitz et al., 2017), feeding from the feeding tube, stationing on the terracotta flower pot, and luring the animal five times to the left and right TT according to a pseudorandom protocol (Gellermann, 1933). Once these pretraining steps were completed, a preference test consisting of a maximum of 10 trials per individual was conducted to reveal whether the animals had a preexisting preference for the left or right side of the aquarium. The location in space marked by a TT preferred by the individual was defined as negative stimulus (S–) during the acquisition phase of the experiment (R0) in which the animal had to learn to only choose one side/one TT (positive stimulus, S+) to get a reward. Upon reaching the learning criterion defined as a performance of ≥80% correct choices (*p* < 0.01, χ^2^ test) in two consecutive sessions of 20 trials, the signs of the stimuli and thus the reward contingencies were reversed; reversal 1 (R1) started. Now the animal had to move to the TT, which had been defined as S– in the previous phase of the experiment, to get a reward. As we conducted a serial reversal learning experiment, every time the animal met the learning criterion, a new reversal (R2, R3–Rn) was initiated until the animal stopped cooperation, most likely due to senescence. Thus, the number of reversals conducted per animal varied.

During all stages of reversal training, the animal started a trial by approaching and sitting on the flower pot (Supplementary Video 1). Subsequently, the monitor was lit, and a 3-min time interval begun during which the animal had to make a decision for the left or right TT. A decision was defined as the animal touching a TT with at least one arm. Dimming of the monitor served as end-of-trial signal upon which the animal's task was to return to the start location. If the animal did not respond to the start signal in the 3-min time interval, the trial was ended. If five trials had to be ended without any response from the animal, the whole session was ended.

The feedback after a response was different for groups 1 and 2. It was varied to study the effect of the ICS. For group 1 including three individuals (Ov1–Ov3), training was started without the ICS, but with positive reinforcement alone. Thus, a correct response was signaled by the secondary reinforcer and food, and an incorrect response was signaled by dimming the monitor directly after the response. As training progressed, the ICS was introduced during R0 in session 40 for Ov1 and Ov2; just to mention for completion, training with the ICS started during R1 in session 15 for Ov3 (see Supplementary Material). At these experimental stages, the animals did not show any sign of learning; moreover, their cooperation was very low. We therefore started ICS signaling, predicting that the feedback after an incorrect response would facilitate the learning process. For group 2 including four individuals (Ov4–Ov7), trained after we had worked with individuals of group 1, incorrect responses were signaled by the immersion of the ICS from the first trial/session during R0 on predicting that, with ICS signaling right from the start of the training, the octopus individuals would continuously learn. Octopus individuals were randomly assigned to one of the two groups.

### Analysis

We analyzed the performance of each animal regarding (1) the number of errors (error referring to an incorrect trial) needed to learn the original task in R0, (2) the number of reversals conducted over the course of the study, (3) the minimum number of errors reached within each group, and (4) the presence of progressive improvement over reversals; these results are reported descriptively. Furthermore progressive improvement was also statistically assessed for every individual, and/or for groups 1 and 2 by averaging the performance of the individuals. For the analysis of progressive improvement, we conducted a linear regression analysis, testing the null hypothesis that the slope of the linear regression is zero. Statistical analyses were run in R 3.3.3 (The R Foundation for Statistical Computing, Vienna, Austria).

## Results

**Figure 2:**
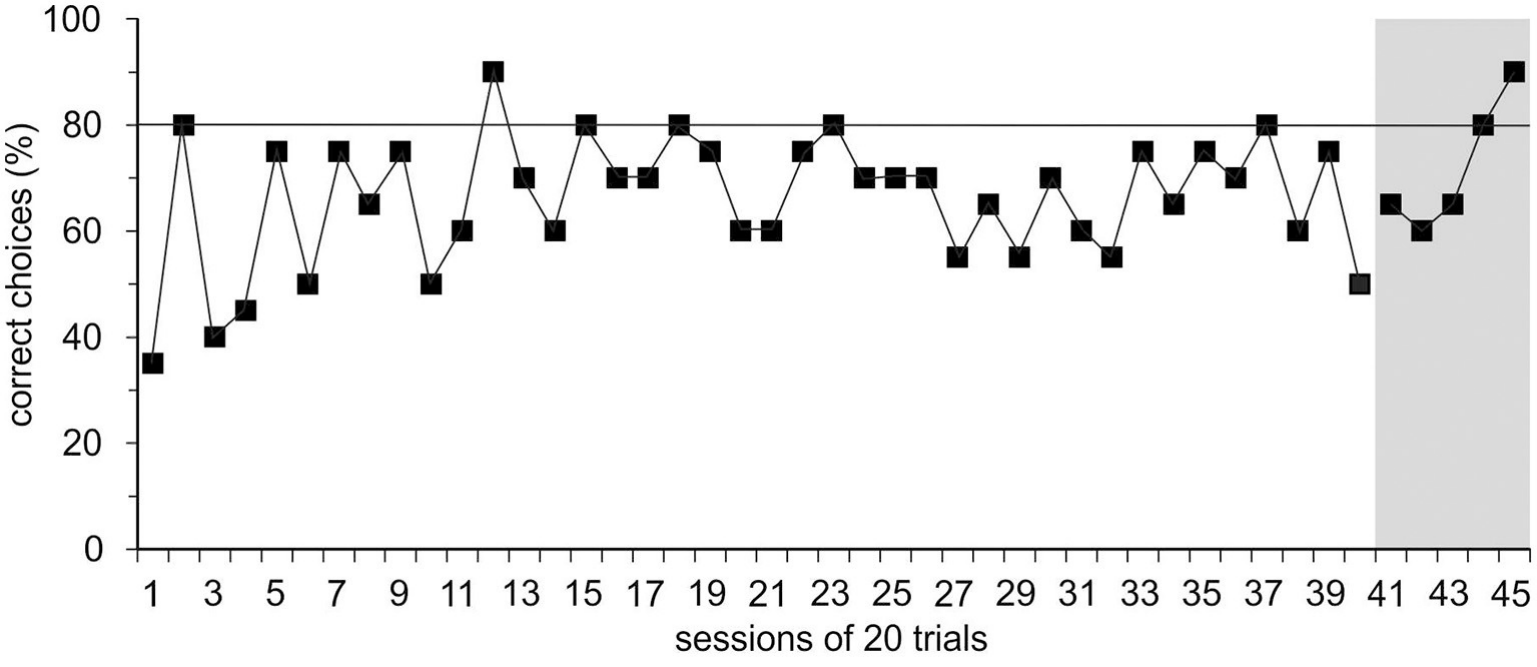
Results of the spatial reversal learning experiment. Exemplary performance of experimental animal Ov1 as correct choices (in %) over time during the acquisition phase (R0) in which it was trained only with positive reinforcement but without incorrect-choice signal (ICS). Ov1 did not reach the learning criterion defined as a performance of ≥80% correct choices in two consecutive sessions (continuous line) in 40 sessions with 20 trials. After the introduction of the ICS indicating the incorrectness of the response in session 41, Ov1, however, reached the learning criterion after five sessions.

In group 1, trained with positive reinforcement alone initially, Ov1 and Ov2 did not learn the basic task; the learning criterion was not met within 40 sessions or after 272 and 346 errors, respectively. Learning stagnated, and the animals hardly cooperated for experiments. With the introduction of the ICS, a black rod signaling an incorrect response, in session 40, R0 could be finished with Ov1 and Ov2 within five and seven sessions or with 38 and 47 errors, respectively (Figure 2).

**Figure 3:**
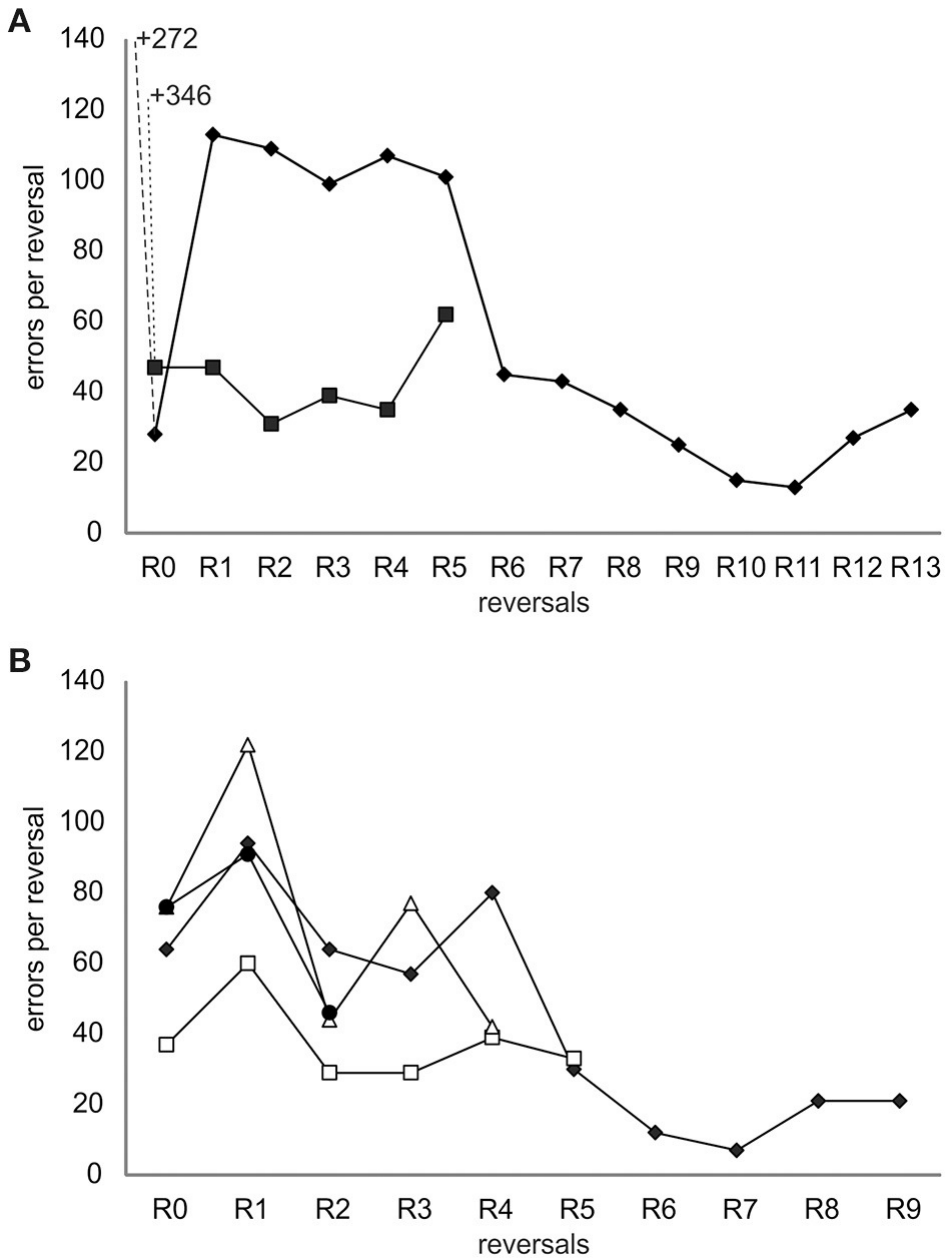
Error curves of all individuals trained in the spatial reversal learning experiment. **(A)** Results from individuals from group 1 trained with the incorrect-choice-signal (ICS) at a later stage of training. The data points indicate the number of errors made until the learning criterion was met during a reversal including the errors made in the two sessions in which it achieved a performance at or exceeding 80% correct choices. The number of errors before ICS signaling was started is written as numbers in the graph allowing the same scaling of the y-axis of the two graphs and thus a direct and better comparison of the performance of groups 1 and 2. The data of Ov1 are marked with filled diamonds and that of Ov2 with filled squares. **(B)** Results from individuals of group 2 trained with the ICS from the beginning of the experiment. The data of Ov4 are marked with filled diamonds, that of Ov5 with open squares, that of Ov6 with open triangles, and that of Ov7 with filled circles. Irrespective of the group, all animals learned the original task and reversed multiple times completing 2–13 reversals, and their performance showed a general trend to improve over time.

After the introduction of the ICS, all individuals finished a number of reversals (Figure 3A and Table 1): Ov1 completed 13 reversals and Ov2, five reversals. Over reversals, Ov1 increased its performance (F-statistics; Ov1 *F* = 44.1, *df* = 11, *p* < 0.01), making fewer errors per reversal the more reversals it experienced. In contrast, Ov2 did not drastically improve its performance over reversals (F-statistics; Ov2 *F* = 0.7, *df* = 3, *p* = 0.46); the number of errors even increased during the last reversal; as the animal stopped cooperating completely thereafter, we assumed that its performance in its last completed reversal had already been caused by a cease in motivation as usually occurring at a late point in octopus' training. Grouping all results, the number of errors decreased significantly over reversal for the individuals of group 1 (F-statistics; Ov1-2 *F* = 9.77, *df* = 16, *p* < 0.01). The minimum number of errors reached by Ov1 and Ov2 was 13 errors in R11. Please note that the results of Ov3 are not included here but in the supplement (see Supplementary Material) due to a slight deviation in training.

**Table 1:** Overview of the performance of the experimental animals during the various phases of the reversal learning experiment depicted as number of errors per reversal.

	**R0**		**R1**	**R2**	**R3**	**R4**	**R5**	**R6**	**R7**	**R8**	**R9**	**R10**	**R11**	**R12**	**R13**
Ov1	272^b^	28^a^	113	109	99	107	101	45	43	35	25	15	13	27	35
	40	5	13	13	11	13	11	5	7	5	4	3	3	5	6
Ov2	346^b^	47^a^	47	31	39	35	62								
	40	4	6	6	5	6	10								
Ov3	**See** **Supplementary Material**														
Ov4	64	94	64	57	80	30	12	7	21	21					
	10	13	11	9	15	7	3	2	3	3					
Ov5	37	60	29	29	39	33									
	7	8	5	5	5	6									
Ov6	76	122	44	77	42										
	13	16	8	11	6										
Ov7	76	91	46												
	10	10	7												

*The number of 20 trials-sessions are indicated below the number of errors for each octopus. For the octopus individuals of group 1 (Ov1–Ov2; results of Ov3 are not displayed here but can be found in the supplements due to a slight difference in training) experiencing the incorrect-choice signal (ICS) marking an incorrect response at a later stage of training, the number of trials conducted before (b) and after the introduction of the ICS signaling an incorrect response (a) are indicated separately for the phase in which the introduction of the ICS took place*.

In group 2, trained with the ICS right from the beginning, Ov4 and Ov7 completed R0 successfully after 10 sessions, Ov5 needed 7 sessions, and Ov6 13 sessions for the completion of R0 (Figure 3B, Table 1). In this group, the individuals were also able to finish numerous reversals thereafter: Ov4 completed nine reversals, Ov5 five reversals, and Ov6 four reversals. Ov7 finished two reversals, and its training had to be stopped in R3 due to a cessation of cooperation from the side of the animal. In this group, the best performance of seven errors per reversal was shown by Ov4 in R7. In general, the performance of all animals increased during R1 and, despite some fluctuations, tended to generally decrease over reversals in the subsequent reversals (F-statistics; Ov4–Ov7 *F* = 18.6, *df* = 18, *p* < 0.01).

## Discussion

In this study, we conducted a spatial serial reversal learning experiment and could show that individuals of the species *O. vulgaris* are able to reverse a simple spatial discrimination task up to 13 times successfully. Some octopus individuals showed clear progressive improvement reaching a performance of 20–30 errors per reversal. The best performance achieved was seven errors to complete a reversal (Ov4 in R7).

The number of errors reached during a reversal in this serial reversal learning experiment including a simple spatial discrimination task was in the same range as for other animals (see, for example, Doty and Combs, 1969; Mackintosh and Cauty, 1971). At the same time, octopus is outperformed by some species (see, for example, Doty and Combs, 1969) also including invertebrates such as the American cockroach (Balderrama, 1980); the cockroaches reached one-trial learning in a reversal learning study including an olfactory discrimination. Among those invertebrates, *S. officinalis*, another cephalopod species, also reduced its errors to one or two errors (Karson et al., 2003), thus to less errors than the octopus of this study. However, these interspecific comparisons have to be made with caution, as methodological differences between studies may strongly influence these results. The cuttlefish, for example, were making their responses when avoiding an unpleasant experimental situation; they were fleeing from a chamber in which they could not settle on the ground (Karson et al., 2003). The differences in performance might thus reflect differences in experimental designs as shown in previous studies (for review, Rayburn-Reeves and Moore, 2018).

The results of this study clearly indicate that learning highly depends on the experimental conditions, the context of learning. Initially, the octopus individuals of group 1 did not learn the spatial discrimination task (Ov1, Ov2) or failed to reverse in R1 (Ov3, see Supplementary Material). The application of the ICS signaling an incorrect response changed the learning behavior of octopus systematically; the individuals learned the respective task with ease. In group 1, all individuals irrespective of the onset of signaling with the ICS learned the original task within seven sessions at maximum after the introduction of the ICS. We think that the animals learned the task because the ICS signaled an incorrect response clearly and not as a result of the intensive training before. Our conclusion is based on several facts: prior to signaling with the ICS, (1) no learning was observed, except for one individual, (2) the animals showed a clear drop of motivation and already started to cease or ceased cooperation, and (3) usually, octopus is learning within a couple of sessions, if they learn at all (Messenger et al., 1973). The last aspect is supported by the learning performance of the individuals of group 2; it took all four individuals trained with the ICS right from the beginning only 13 sessions at maximum to solve the original task, and the high variability documented in other studies (see, for example, Bublitz et al., 2017) was not as apparent. Moreover, we have clear evidence from the octopus behavior that they have actually perceived the signal because, upon the introduction of the ICS, they initially/sometimes changed their body pattern to the broad mottle display (Figure 1C; Packard and Sanders, 1971). In conclusion, we think that octopus learning is positively affected by an ICS, an aspect that, however, needs to be investigated in detail.

This conclusion, that learning is positively affected by the ICS, is supported by previous octopus discrimination or learning studies in which very strong feedback for incorrect responses was provided (see, for example, Young, 1961, 1962b; Mackintosh and Mackintosh, 1963). In these experiments, octopus also performed well. The positive effect on learning might occur because an ICS directly indicates an incorrect response. In contrast, using positive reinforcement alone, the incorrectness of a response is only indirectly signaled by the absence of positive feedback or by the absence of food. However, some animals might need an unambiguous feedback even after responding incorrectly (see, for example, honey bees in Avarguès-Weber et al., 2010). If we can generalize the effect of an ICS over experiments, it is still unresolved why octopus training profits from an ICS or, regarding the study at hand, why positive reinforcement alone did not allow most octopus individuals to learn the task. From an ecological perspective, food might not be the sole or even the main driver of octopus behavior, as prey is probably not a limiting factor for a generalist under water (Mather, 1991a; Mather et al., 2012). Octopus might initiate behavioral changes when an external event clearly indicates the inappropriateness of its behavior just shown. Thus, a combination of positive reinforcement and signaling with an ICS might cause learning, as it mimics the natural situation of octopus.

In contrast to previous discrimination or learning studies in octopus, we can show with our training results, that a neutral signal, a black rod, can easily be associated with incorrect responses causing no harm. Even to the contrary, the animals simply detached from the TTs and moved toward the station allowing the next trial to start. It is conceivable for future experiments to use an alternative ICS, such as a looming stimulus on a monitor as described in Pignatelli et al. (2011); however the looming stimulus should be reduced in strength to avoid the strong avoidance responses shown by the cephalopods. Preliminary results from our training indicate that this signal could be equally effective (unpublished results). A visual signal on a monitor would allow standardizing the signal and might be easier to apply depending on the experimental task or setup.

One of our motivations for this study was to contrast the reversal performance of octopus in a visual (Bublitz et al., 2017) versus a spatial discrimination task. Comparing the performance during R0 during visual reversal learning with the performance of group 1 prior to the introduction of the new experimental tool, the ICS, it is directly evident that the visual discrimination was acquired much faster than the spatial discrimination. It took octopus individuals 60–459 trials to learn a visual discrimination (Bublitz et al., 2017) or even less in Mackintosh and Mackintosh (1964). However, only one individual, Ov3, was able to reach the learning criterion within this range of trials, after 100 trials, when trained for a spatial discrimination most likely due to its previous experience (see *Experimental subjects* and Supplementary Material). The other two individuals of group 1 did not even learn the spatial discrimination task within 800 trials. Moreover, compared to the acquisition rates of most octopus in visual experiments, the acquisition rate of the octopus from group 2, which were, however, trained with the ICS, was slower than in the visual experiments. In conclusion, a spatial discrimination task does not seem to be easier to solve for an octopus than a visual task. This finding is contrary to our expectation that was based on theoretical and empirical considerations (see *Introduction*), suggesting that octopuses have good spatial skills leading to good spatial discrimination abilities. Good and maybe even better visual abilities, on the other hand, fit to the well-developed visual system and the high neuronal investment for the processing of visual stimuli in the large optic lobes of octopus (Young, 1962a; Maddock and Young, 1987; Budelmann, 1994).

Ultimately, spatial serial reversal learning could not be tested with positive reinforcement alone rendering a comparison of reversal performance with spatial versus visual cues difficult. To allow direct comparison, future work should revisit visual reversal learning with a methodology including positive reinforcement and ICS signaling. However, despite differences in experimental design, we can conclude that with both types of tasks, most octopus can learn to reverse multiple times in succession. Octopus can increase its performance over reversals; however, the minimum number of errors per reversal varies across studies (compare with, for example, Bublitz 2017; Mackintosh and Mackintosh, 1964). As octopus is responding flexibly to spatial as well as nonspatial cues, such as visual cues, the selection might have favored behavioral flexibility in octopus, in general, a thought raised by Day et al. (1999). This finding is also in line with the hypothesis that learning and flexibility in handling of previously learned aspects are crucial from the point of view of octopus biology. Factors that possibly require well-developed learning abilities in general and reversal learning abilities in particular are (1) the short lifespan of octopus during which long learning phases can be fatal, thus learning from experience is vital; (2) its active foraging mode during which the animals most likely have to make decisions to familiar and novel stimuli in the same or a new context quickly; (3) competition for niches with other animals; or (4) predator pressure, which is particularly high in a soft-bodied animal (for a detailed discussion, see Bublitz et al., 2017). Flexibility in behavior has previously been shown regarding the presence of predators (Meisel et al., 2013) or the construction of dens (Mather and Dickel, 2017); thus, from an ecological perspective, flexible responding to familiar conditions, as tested during reversal learning, might be essential and indeed occurring in octopus. An interesting avenue for future research could be to test how vision supports spatial orientation allowing the animal to construct a visuospatial map of its home range (Mather, 1991a).

Overall, its cognitive abilities allow the octopus to not only solve a discrimination problem but also to reverse previously learned responses. Thus, octopus can learn more than during discrimination learning, meaning more than the association between a stimulus and its associated response.

## Data Availability Statement

The original contributions presented in the study are included in the article/Supplementary Material, further inquiries can be directed to the corresponding author.

## Ethics Statement

The animal study was reviewed and approved by Staatliches Amt für Umwelt and Natur Rostock, Landesamt für Landwirtschaft, Lebensmittelsicherheit und Fischerei, Mecklenburg-Vorpommern.

## Author Contributions

The study was designed by all authors. AB conducted the experiments, analyzed the data, and wrote the first version of the manuscript. All authors edited the manuscript and approved the final version.

## Conflict of Interest

The authors declare that the research was conducted in the absence of any commercial or financial relationships that could be construed as a potential conflict of interest.
