# Supplementary material for: Reversal of a Spatial Discrimination Task in the Common Octopus (Octopus vulgaris)
Source: Front Behav Neurosci. 2021 Jun 25;15:614523. doi: 10.3389/fnbeh.2021.614523 (PMC8267067; doi:10.3389/fnbeh.2021.614523)

Supplement for

Bublitz et al.

**Reversal of a spatial discrimination task in the common octopus *(Octopus vulgaris)***

Supplements Table 1 Overview of the performance of the experimental animals during the various phases of the reversal learning experiment depicted as number of errors per reversal. The number of 20 trials-session are indicated below the number of errors for each octopus. For the octopus individuals of group 1 (Ov1-3; Ov3 is included here for completion, although its first training steps deviated slightly from the other Ov individuals) experiencing the incorrect-choice signal (ICS) marking an incorrect response at a later stage of training, the number of trials conducted before (b) and after the introduction of the ICS signaling an incorrect response (a) are indicated separately for the phase in which the introduction of the ICS took place, either R0 or R1.

| | R0 | | R1 | | R2 | R3 | R4 | R5 | R6 | R7 | R8 | R9 | R10 | R11 | R12 | R13 |
|---|---|---|---|---|---|---|---|---|---|---|---|---|---|---|---|---|
| Ov1 | 272[b] | 28[a] | 113 | | 109 | 99 | 107 | 101 | 45 | 43 | 35 | 25 | 15 | 13 | 27 | 35 |
| | 40 | 5 | 13 | | 13 | 11 | 13 | 11 | 5 | 7 | 5 | 4 | 3 | 3 | 5 | 6 |
| Ov2 | 346[b] | 47[a] | 47 | | 31 | 39 | 35 | 62 | | | | | | | | |
| | 40 | 4 | 6 | | 6 | 5 | 6 | 10 | | | | | | | | |
| Ov3 | 17 | | 127[b] | 28[a] | 93 | 77 | 71 | 80 | 18 | 18 | 11 | 31 | 20 | | | |
| | 5 | | 15 | 5 | 12 | 10 | 11 | 11 | 3 | 3 | 5 | 3 | 4 | | | |
| Ov4 | 64 | | 94 | | 64 | 57 | 80 | 30 | 12 | 7 | 21 | 21 | | | | |
| | 10 | | 13 | | 11 | 9 | 15 | 7 | 3 | 2 | 3 | 3 | | | | |
| Ov5 | 37 | | 60 | | 29 | 29 | 39 | 33 | | | | | | | | |
| | 7 | | 8 | | 5 | 5 | 5 | 6 | | | | | | | | |
| Ov6 | 76 | | 122 | | 44 | 77 | 42 | | | | | | | | | |
| | 13 | | 16 | | 8 | 11 | 6 | | | | | | | | | |
| Ov7 | 76 | | 91 | | 46 | | | | | | | | | | | |
| | 10 | | 10 | | 7 | | | | | | | | | | | |

Supplements Figure 1

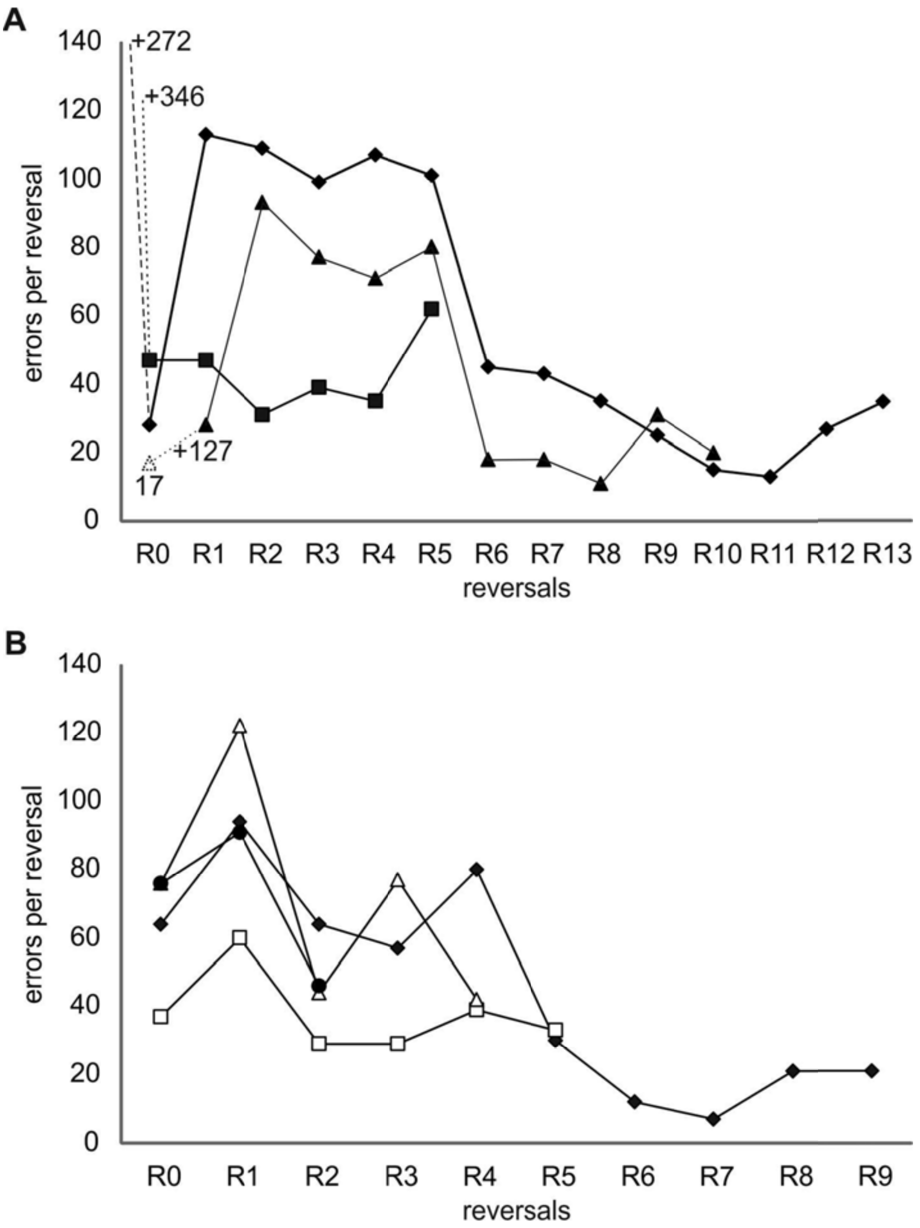


Error curves of all individuals (including Ov3) trained in the spatial reversal learning experiment. **(A)** Results from individuals from group 1 trained with the incorrect-choice-signal (ICS) at a later stage of training, either during R0 or R1. The data points indicate the number of errors made until the learning criterion was met during a reversal including the errors made in the two sessions in which it achieved a performance at or exceeding 80% correct choices. The number of errors before ICS signaling was started are written as numbers in the graph allowing the same scaling of the y-axis of the two graphs and thus a direct and better comparison of the performance of group 1 and group 2. The data of Ov1 are marked with filled diamonds, the data of Ov2 with filled squares, and the data of Ov3 with filled triangles. **(B)** Results from individuals of group 2 trained with the ICS from the beginning of the experiment. The data of Ov4 are marked with filled diamonds, the data of Ov5 with open squares, the data of Ov6 with open triangles, and the data of Ov7 with filled circles. All animals irrespective of the group learnt the original task, reversed multiple times completing 2-13 reversals, and their performance also showed a general trend to improve over time irrespective of the group.

Learning performance of Ov1

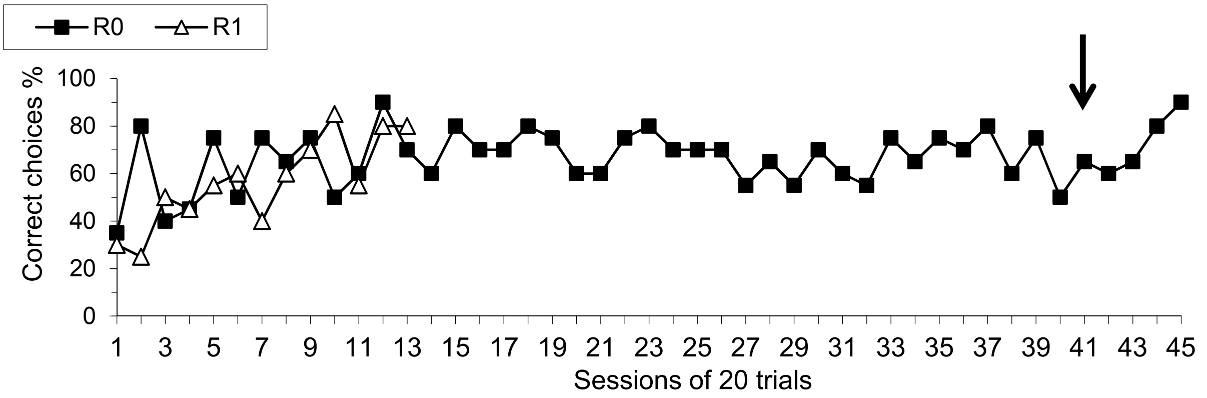


Arrow marks introduction of ICS (see text for details)

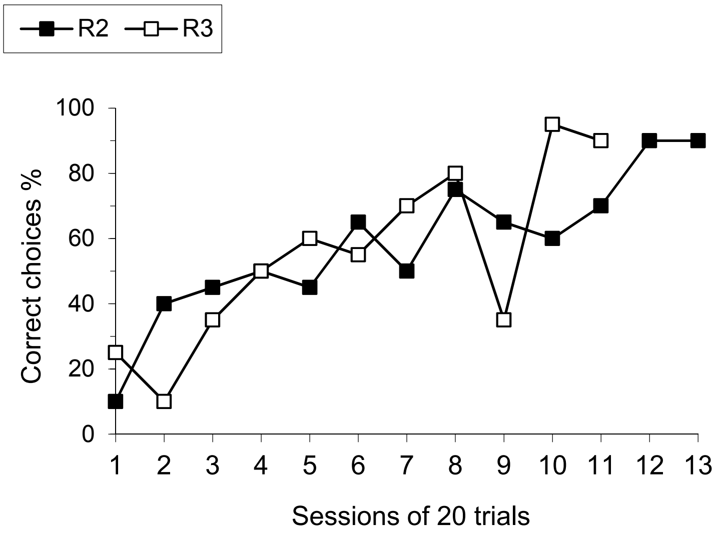


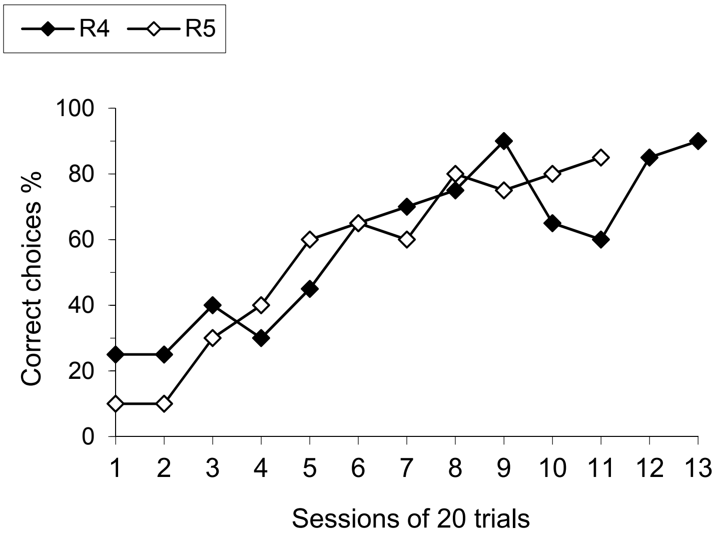

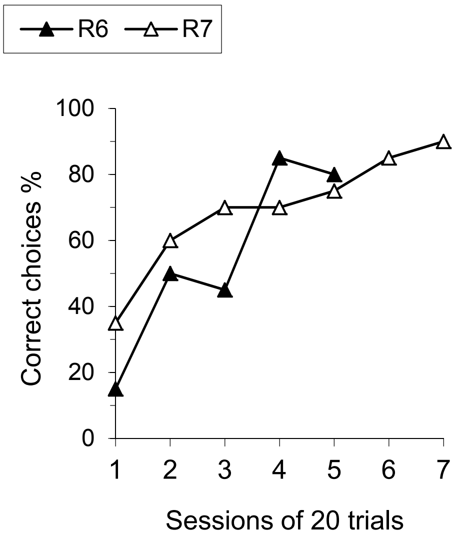
R6
R7
Correct choices %
0
20
40
60
80
100
1
2
3
4
5
6
7
Sessions of 20 trials

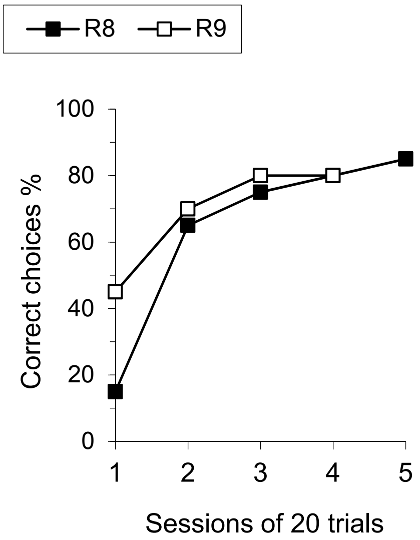
R8
R9
Correct choices %
0
20
40
60
80
100
1
2
3
4
5
Sessions of 20 trials

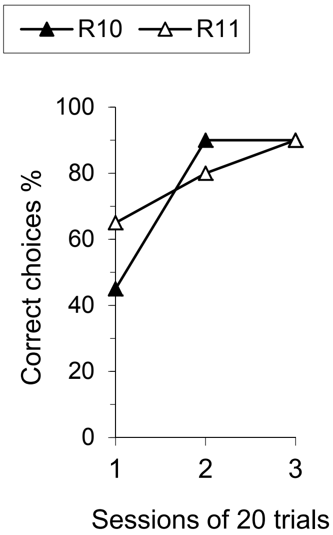
R10
R11
Correct choices %
0
20
40
60
80
100
1
2
3
Sessions of 20 trials

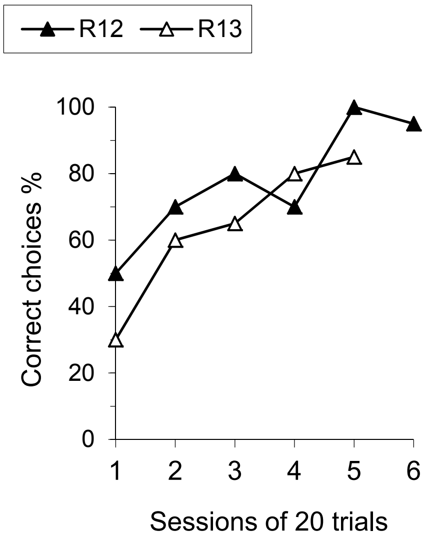
R12
R13
Correct choices %
0
20
40
60
80
100
1
2
3
4
5
6
Sessions of 20 trials

Learning performance of Ov2

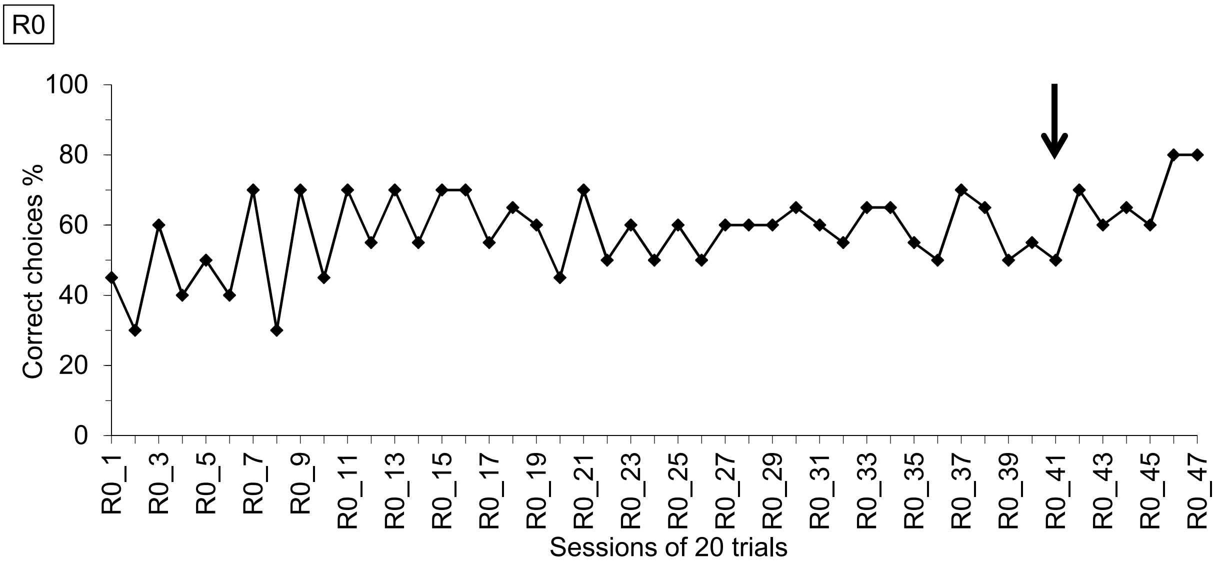


Arrow marks introduction of ICS (see text for details)

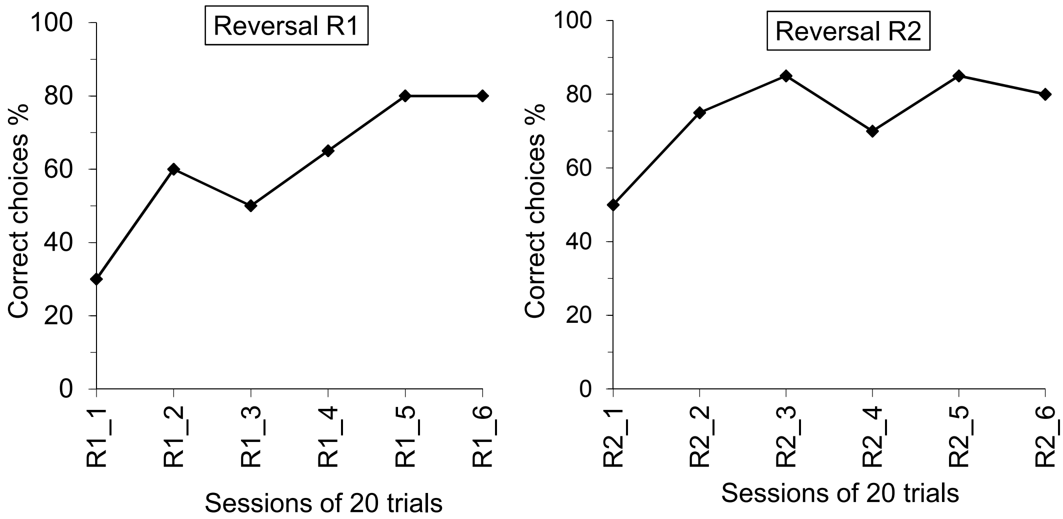

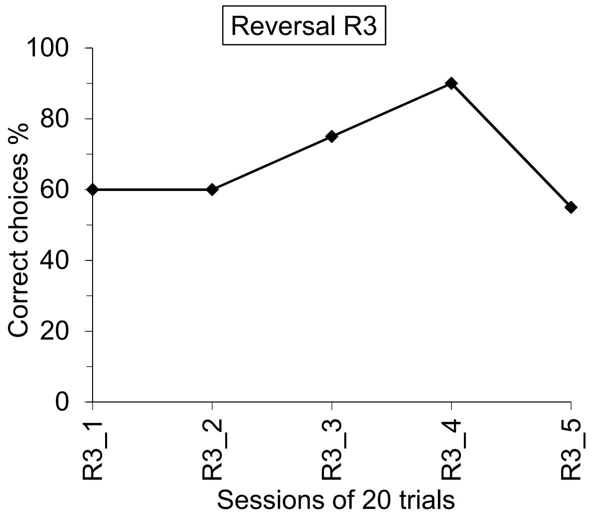
Reversal R3
Correct choices %
100
80
60
40
20
0
R3_1
R3_2
R3_3
R3_4
R3_5
Sessions of 20 trials

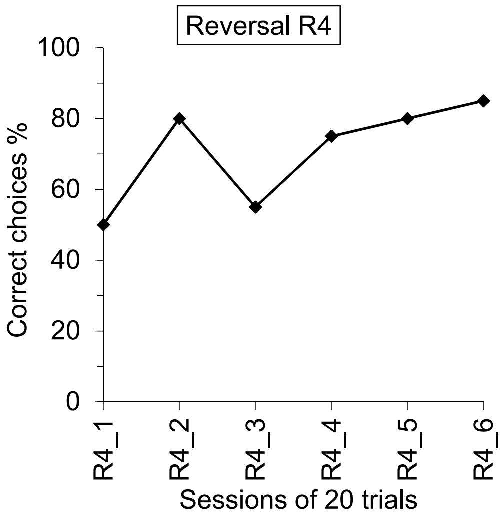
Reversal R4
Correct choices %
100
80
60
40
20
0
R4_1
R4_2
R4_3
R4_4
R4_5
R4_6
Sessions of 20 trials

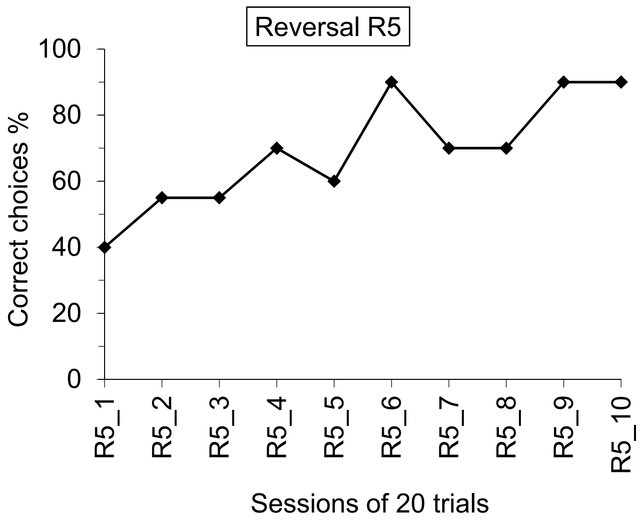
Reversal R5
Correct choices %
100
80
60
40
20
0
R5_1
R5_2
R5_3
R5_4
R5_5
R5_6
R5_7
R5_8
R5_9
R5_10
Sessions of 20 trials

Learning performance of Ov3

Ov3 increased its performance over reversals (F=18.2, df=7, p<0.01).

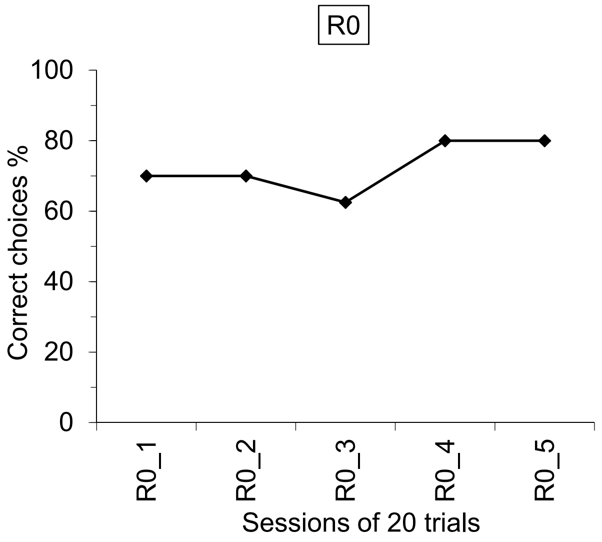


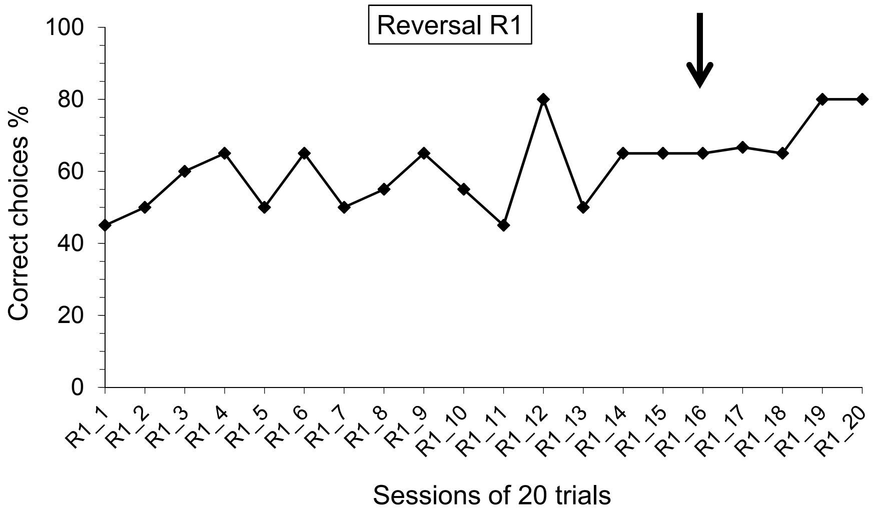


Arrow marks introduction of ICS (see text for details)

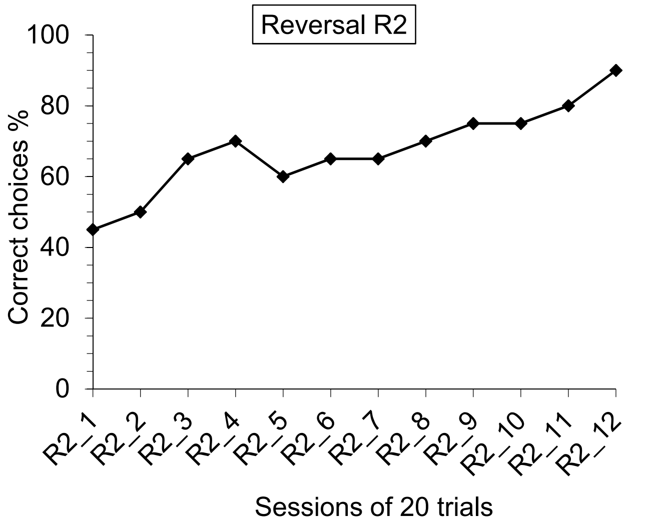
Reversal R2
Correct choices %
100
80
60
40
20
0
R2_1
R2_2
R2_3
R2_4
R2_5
R2_6
R2_7
R2_8
R2_9
R2_10
R2_11
R2_12
Sessions of 20 trials

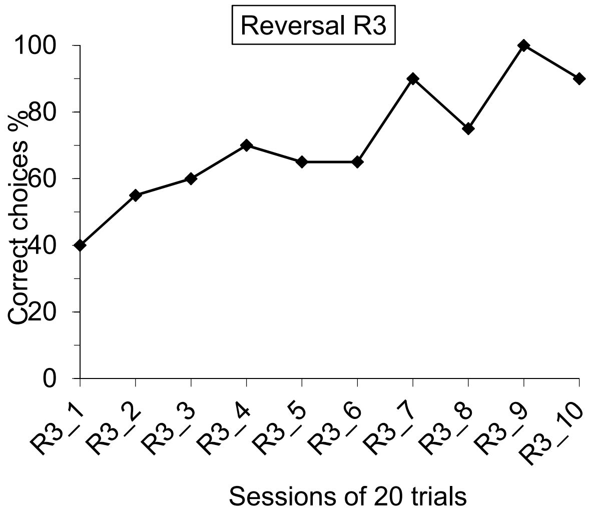
Reversal R3
Correct choices %
100
80
60
40
20
0
R3_1
R3_2
R3_3
R3_4
R3_5
R3_6
R3_7
R3_8
R3_9
R3_10
Sessions of 20 trials

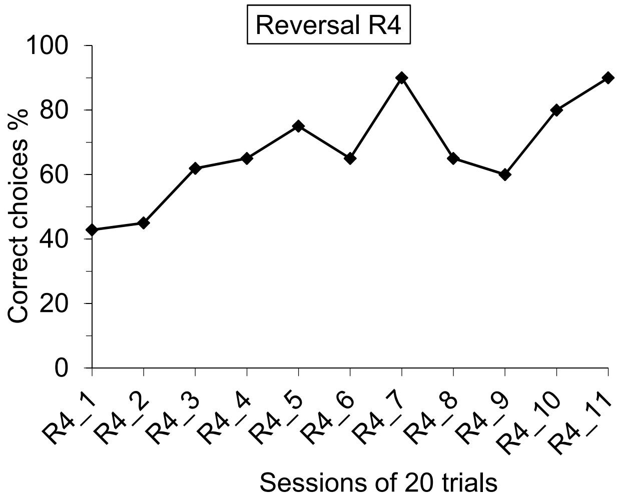
Reversal R4
Correct choices %
100
80
60
40
20
0
R4_1
R4_2
R4_3
R4_4
R4_5
R4_6
R4_7
R4_8
R4_9
R4_10
R4_11
Sessions of 20 trials

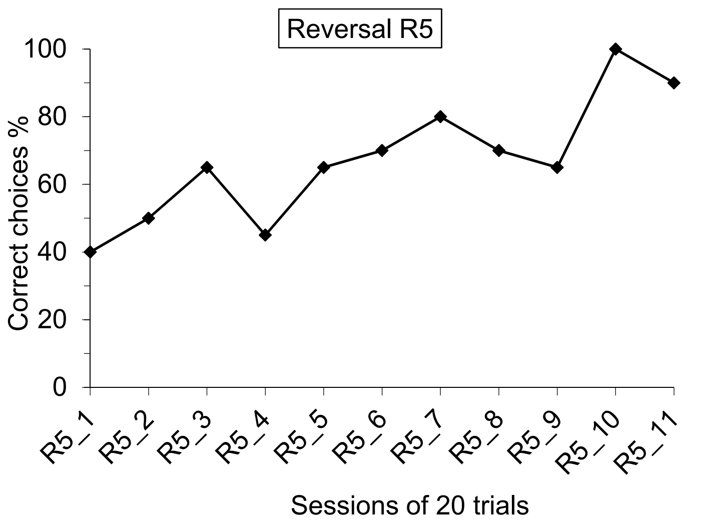
Reversal R5
Correct choices %
0
20
40
60
80
100
R5_1
R5_2
R5_3
R5_4
R5_5
R5_6
R5_7
R5_8
R5_9
R5_10
R5_11
Sessions of 20 trials

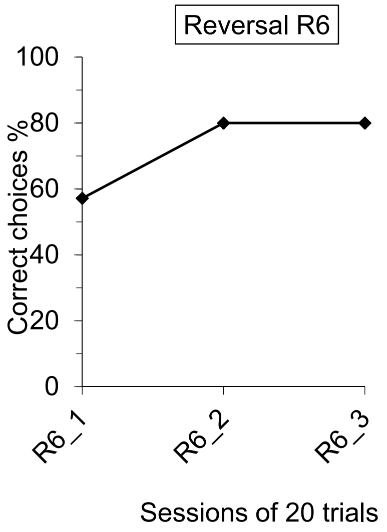
Reversal R6
Correct choices %
0
20
40
60
80
100
R6_1
R6_2
R6_3
Sessions of 20 trials

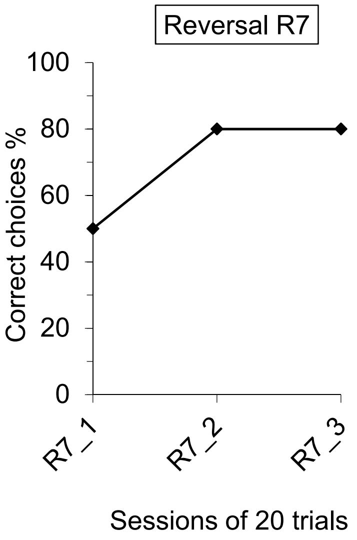
Reversal R7
Correct choices %
0
20
40
60
80
100
R7_1
R7_2
R7_3
Sessions of 20 trials

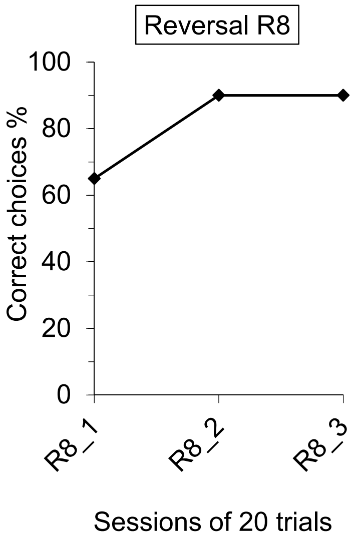
Reversal R8
Correct choices %
0
20
40
60
80
100
R8_1
R8_2
R8_3
Sessions of 20 trials

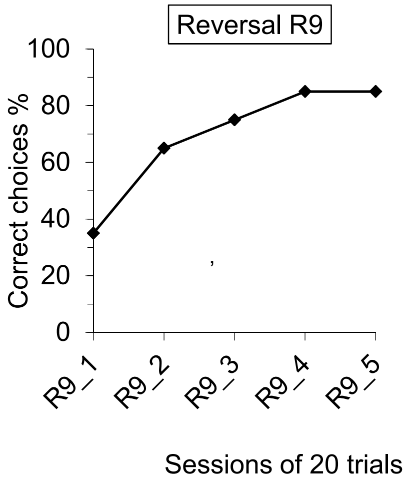
Reversal R9
Correct choices %
0
20
40
60
80
100
R9_1
R9_2
R9_3
R9_4
R9_5
Sessions of 20 trials

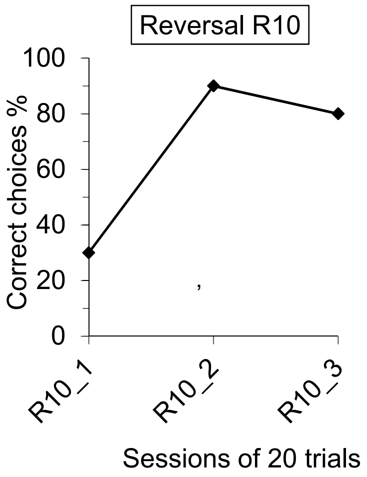
Reversal R10
Correct choices %
0
20
40
60
80
100
R10_1
R10_2
R10_3
Sessions of 20 trials

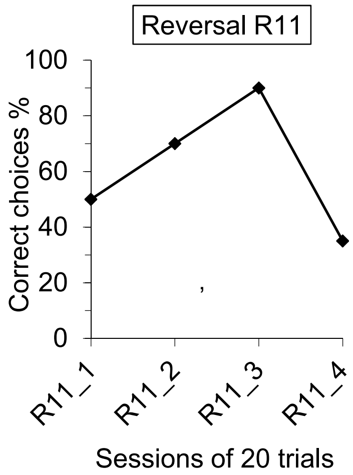
Reversal R11
Correct choices %
0
20
40
60
80
100
R11_1
R11_2
R11_3
R11_4
Sessions of 20 trials

Learning performance of Ov4

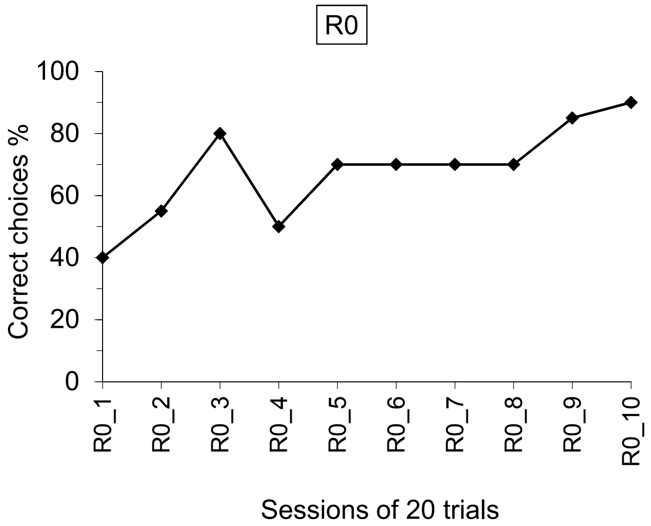


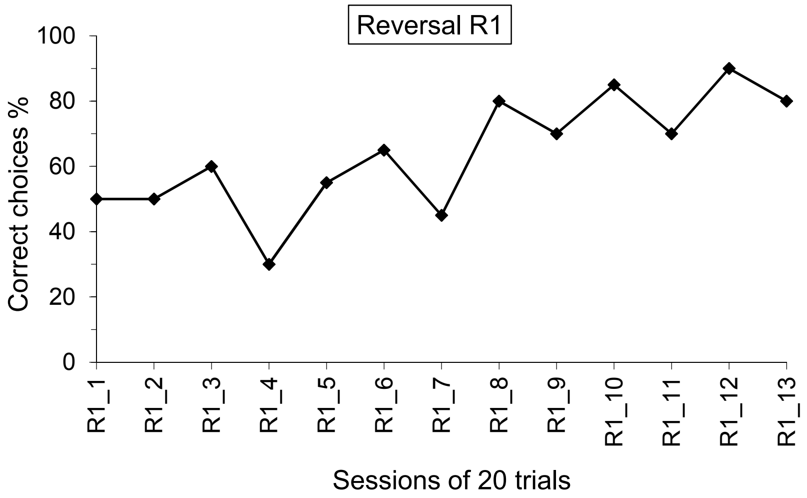


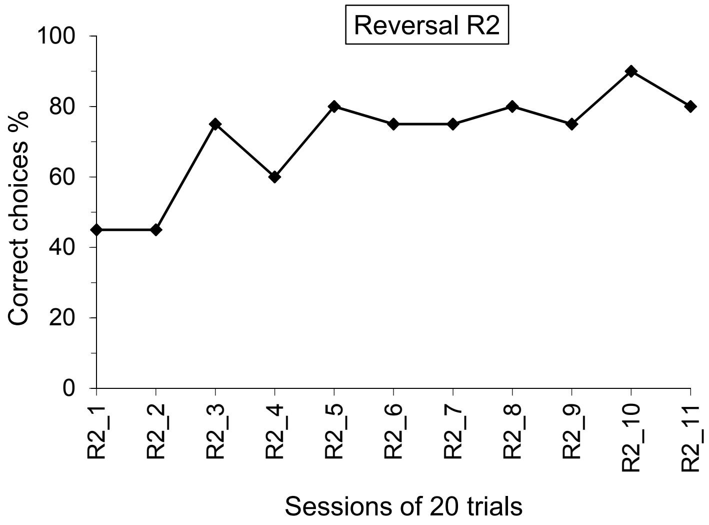

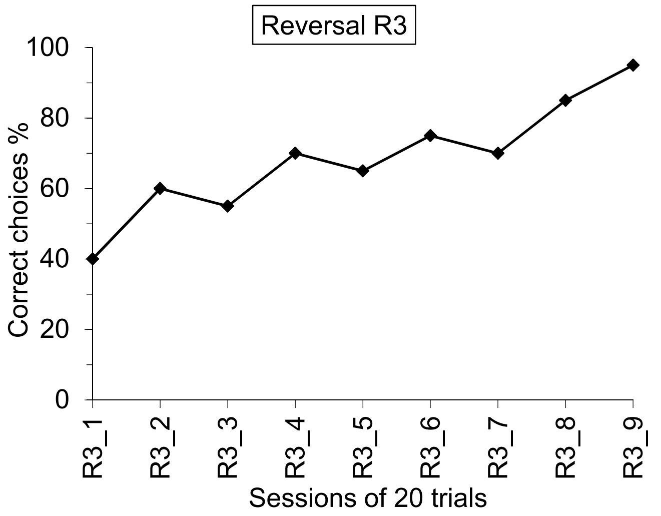
Reversal R3
Correct choices %
100
80
60
40
20
0
R3_1
R3_2
R3_3
R3_4
R3_5
R3_6
R3_7
R3_8
R3_9
Sessions of 20 trials

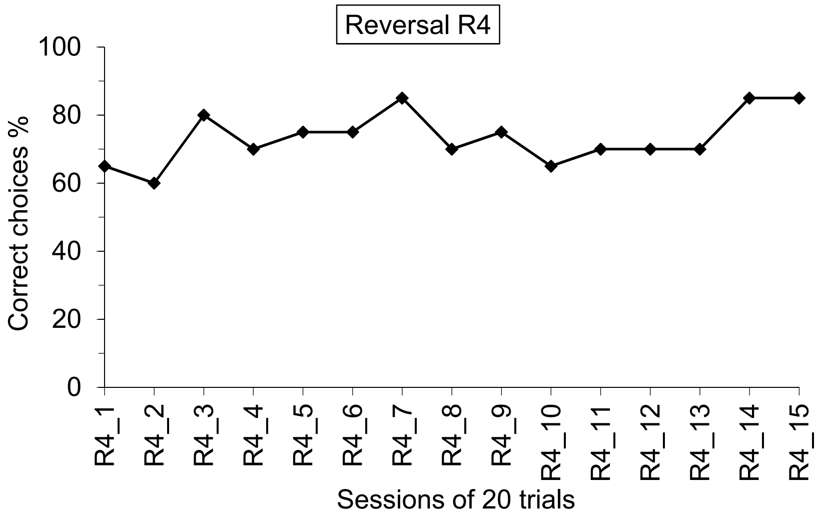
Reversal R4
Correct choices %
100
80
60
40
20
0
R4_1
R4_2
R4_3
R4_4
R4_5
R4_6
R4_7
R4_8
R4_9
R4_10
R4_11
R4_12
R4_13
R4_14
R4_15
Sessions of 20 trials

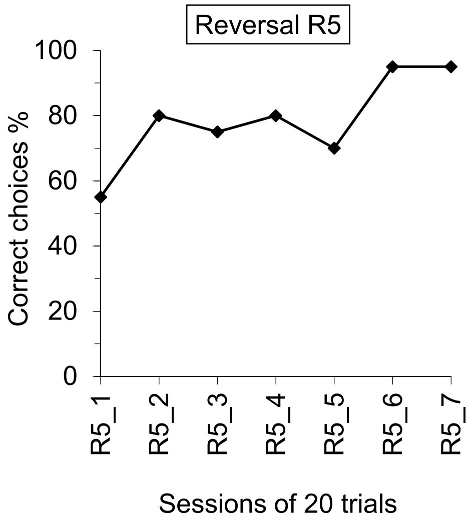
Reversal R5
Correct choices %
100
80
60
40
20
0
R5_1
R5_2
R5_3
R5_4
R5_5
R5_6
R5_7
Sessions of 20 trials

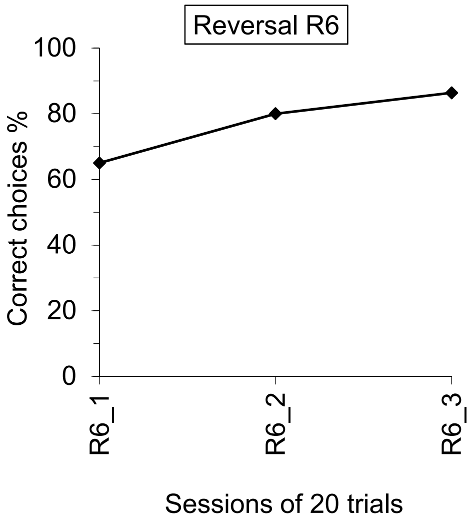
Reversal R6
Correct choices %
100
80
60
40
20
0
R6_1
R6_2
R6_3
Sessions of 20 trials

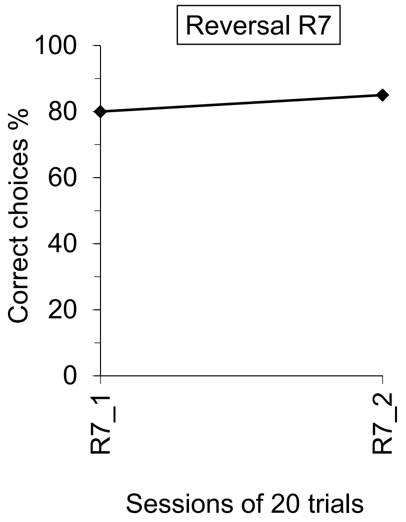
Reversal R7
Correct choices %
100
80
60
40
20
0
R7_1
R7_2
Sessions of 20 trials

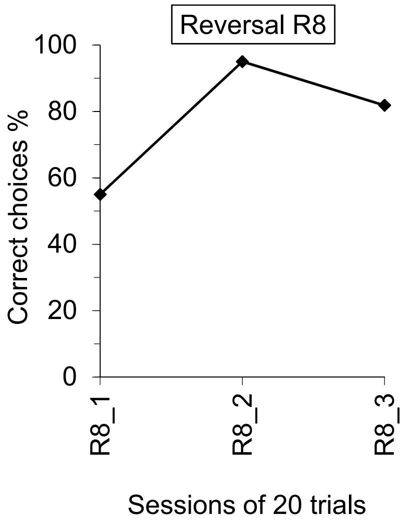
Reversal R8
Correct choices %
100
80
60
40
20
0
R8_1
R8_2
R8_3
Sessions of 20 trials

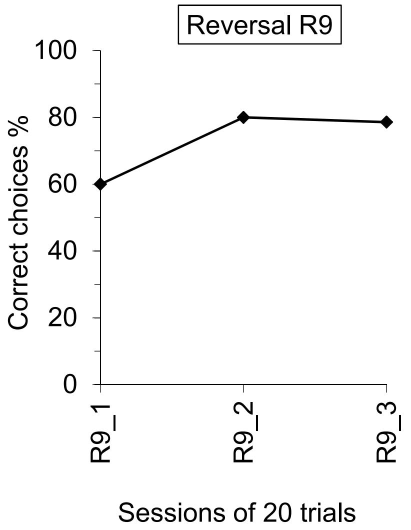
Reversal R9
Correct choices %
100
80
60
40
20
0
R9_1
R9_2
R9_3
Sessions of 20 trials

Learning performance of Ov5

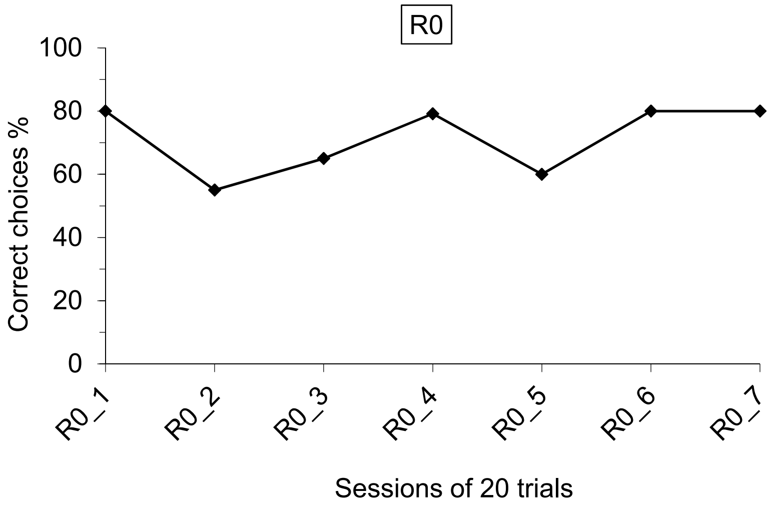


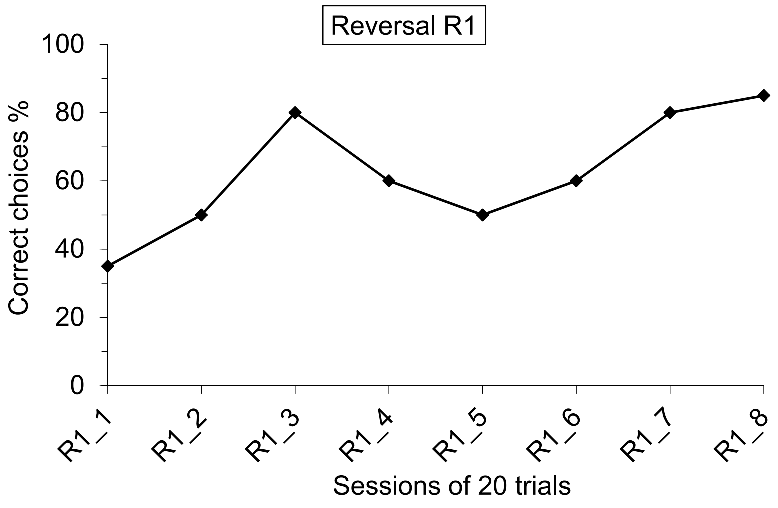


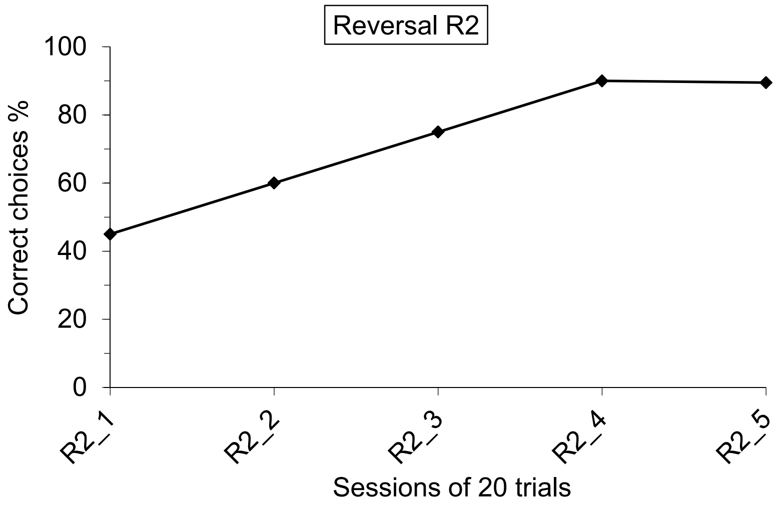

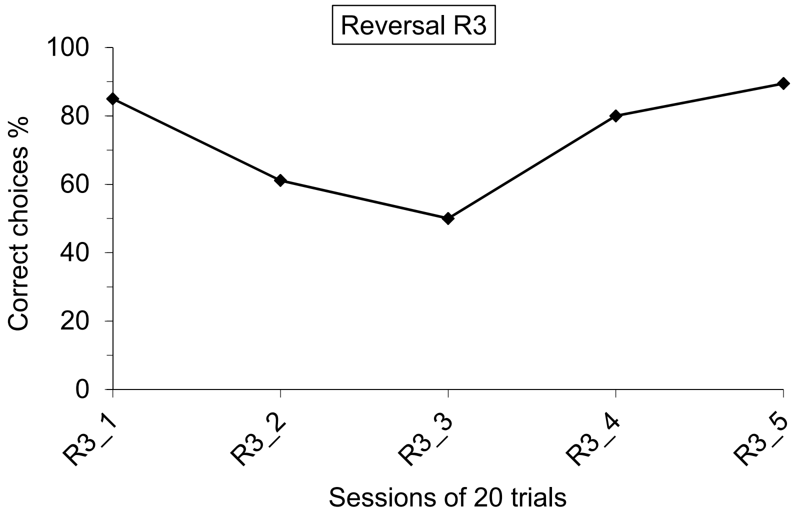
Reversal R3
Correct choices %
100
80
60
40
20
0
R3_1
R3_2
R3_3
R3_4
R3_5
Sessions of 20 trials

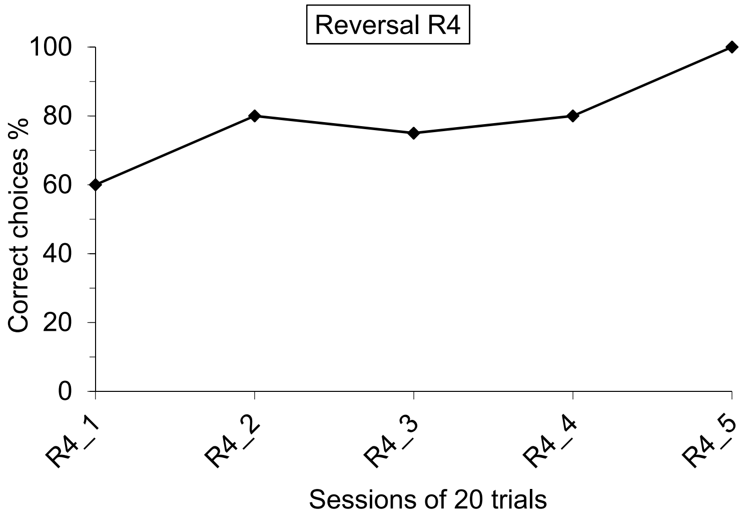
Reversal R4
Correct choices %
100
80
60
40
20
0
R4_1
R4_2
R4_3
R4_4
R4_5
Sessions of 20 trials

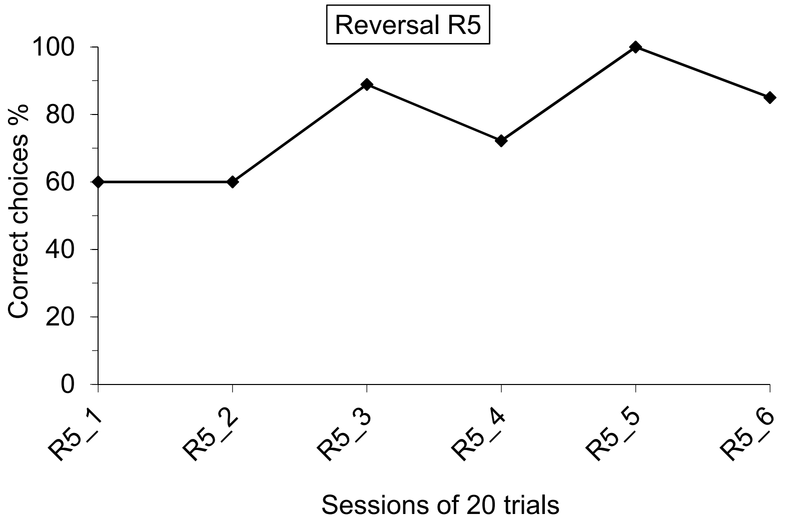
Reversal R5
Correct choices %
100
80
60
40
20
0
R5_1
R5_2
R5_3
R5_4
R5_5
R5_6
Sessions of 20 trials

Learning performance of Ov6

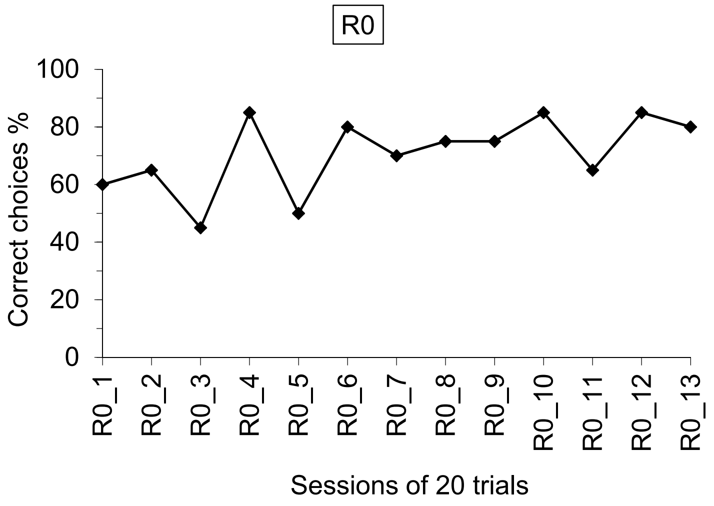


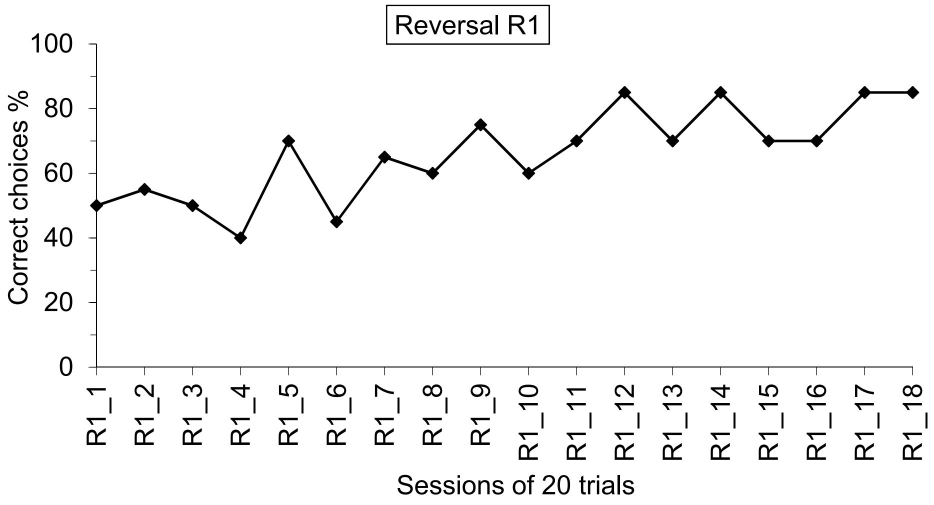

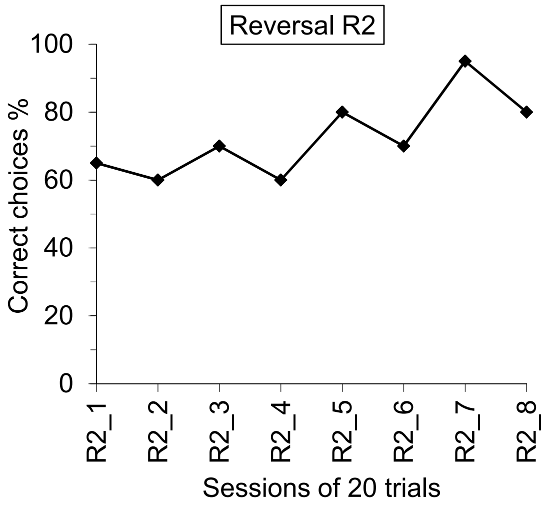
Reversal R2
Correct choices %
0
20
40
60
80
100
R2_1
R2_2
R2_3
R2_4
R2_5
R2_6
R2_7
R2_8
Sessions of 20 trials

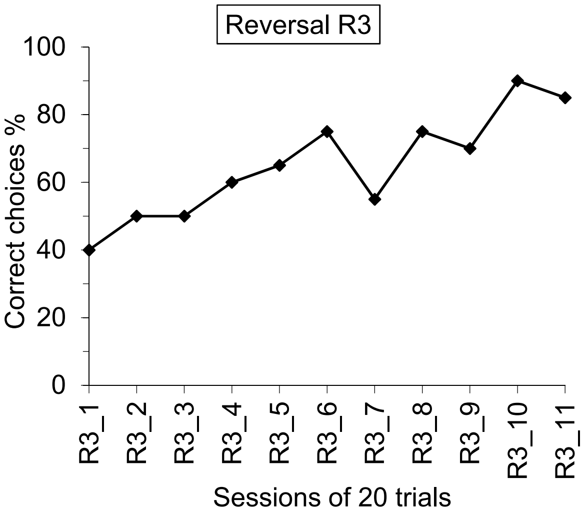
Reversal R3
Correct choices %
0
20
40
60
80
100
R3_1
R3_2
R3_3
R3_4
R3_5
R3_6
R3_7
R3_8
R3_9
R3_10
R3_11
Sessions of 20 trials

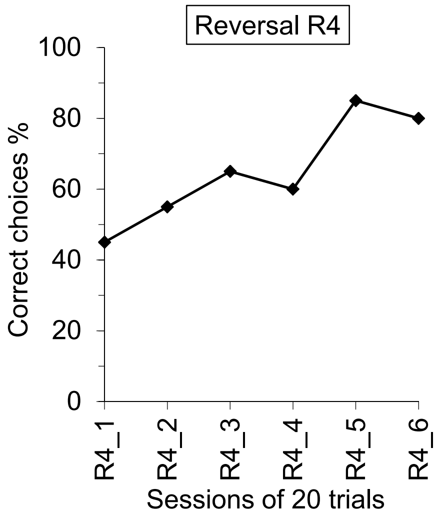
Reversal R4
Correct choices %
0
20
40
60
80
100
R4_1
R4_2
R4_3
R4_4
R4_5
R4_6
Sessions of 20 trials

Learning performance of Ov7

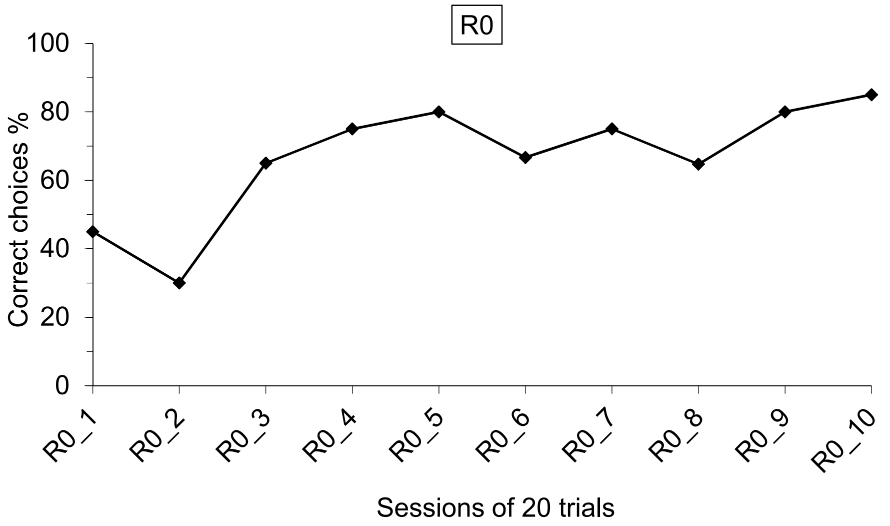


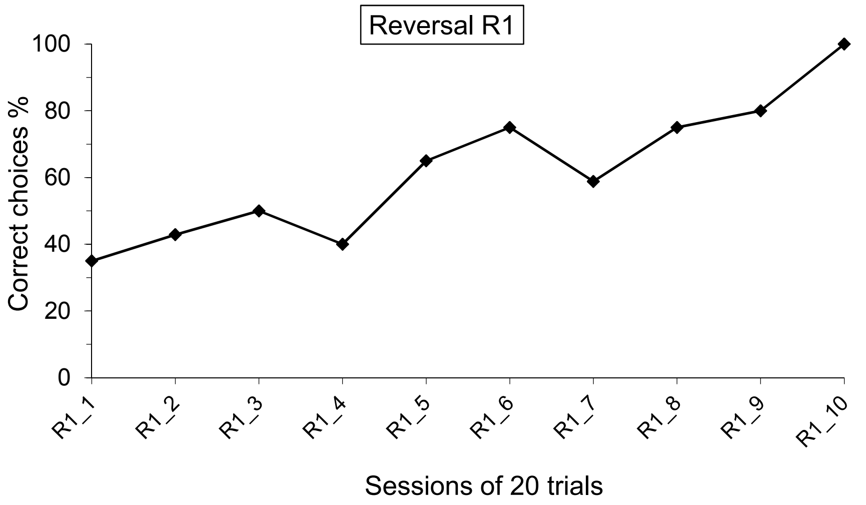


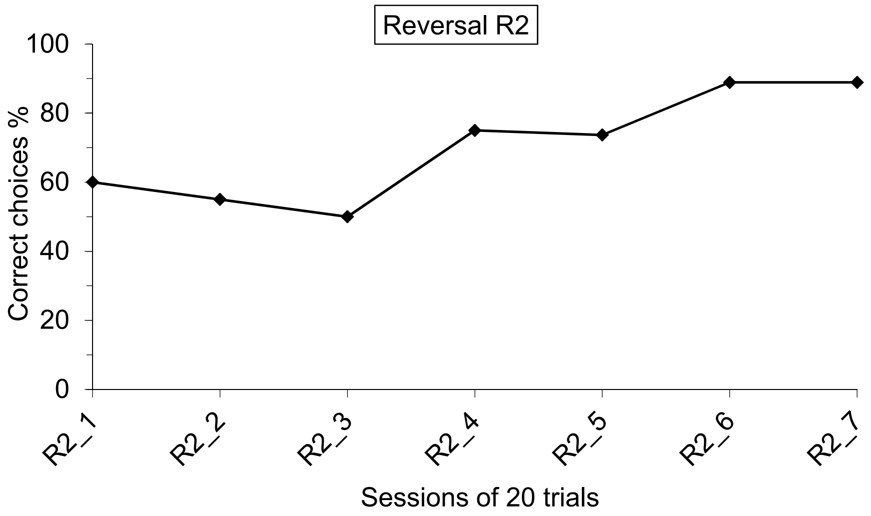

Supplement: Supplementary file 1 [file Data_Sheet_1.PDF]
